# Research on hybrid reservoir scheduling optimization based on improved walrus optimization algorithm with coupling adaptive ε constraint and multi-strategy optimization

**DOI:** 10.1038/s41598-024-62722-8

**Published:** 2024-05-25

**Authors:** Ji He, Yefeng Tang, Xiaoqi Guo, Haitao Chen, Wen Guo

**Affiliations:** College of Water Resources, Henan Key Laboratory of Water Resources Conservation and Intensive Utilization in the Yellow River Basin, North China University of Water Resources and Electric Power, Zhengzhou, 450046 China

**Keywords:** ε-IWOA Algorithm, Luanhe river basin, Hybrid reservoir group, Flood control storage capacity, Ecology, Environmental sciences, Hydrology, Natural hazards

## Abstract

Reservoir flood control scheduling is a challenging optimization task, particularly due to the complexity of various constraints. This paper proposes an innovative algorithm design approach to address this challenge. Combining the basic walrus optimization algorithm with the adaptive ε-constraint method and introducing the SPM chaotic mapping for population initialization, spiral search strategy, and local enhancement search strategy based on Cauchy mutation and reverse learning significantly enhances the algorithm's optimization performance. On this basis, innovate an adaptive approach ε A New Algorithm for Constraints and Multi Strategy Optimization Improvement (ε-IWOA). To validate the performance of the ε-IWOA algorithm, 24 constrained optimization test functions are used to test its optimization capabilities and effectiveness in solving constrained optimization problems. Experimental results demonstrate that the ε-IWOA algorithm exhibits excellent optimization ability and stable performance. Taking the Taolinkou Reservoir, Daheiting Reservoir, and Panjiakou Reservoir in the middle and lower reaches of the Luanhe River Basin as a case study, this paper applies the ε-IWOA algorithm to practical reservoir scheduling problems by constructing a three-reservoir flood control scheduling system with Luanxian as the control point. A comparative analysis is conducted with the ε-WOA, ε-DE and ε-PSO (particle swarm optimization) algorithms.The experimental results indicate that ε-IWOA algorithm performs the best in optimization, with the occupied flood control capacity of the three reservoirs reaching 89.32%, 90.02%, and 80.95%, respectively. The control points in Luan County can reduce the peak by 49%.This provides a practical and effective solution method for reservoir optimization scheduling models. This study offers new ideas and solutions for flood control optimization scheduling of reservoir groups, contributing to the optimization and development of reservoir scheduling work.

## Introduction

Flood disasters are currently the most frequent natural disasters, with statistics showing that there were 109 flood disasters globally in 2018 alone, resulting in 1,995 deaths and affecting 12.62 million people. Direct economic losses amounted to USD 4.5 billion. Among these, China, India, Indonesia, and the United States are the countries that have experienced the highest number of flood disasters and suffered the most significant losses^1^.To reduce the harm caused by flood disasters to people's lives and property, it is necessary to regulate the water resources within a region^2^. This process is known as flood control scheduling, which involves the real-time planning of floodwater using flood control engineering or aquatic structures to achieve maximum flood control benefits^3,4^.

Reservoirs serve as crucial engineering measures that regulate and store floodwater, reduce flood peak flows, and ensure the safety of downstream areas^5^. As an effective non-engineering measure to address flood disasters, flood control optimization and regulation of reservoirs has always been a research hotspot. This is because it can precisely coordinate the flood control efforts of various reservoir groups, thereby safeguarding the flood control safety of the entire river basin^6,7^. However, as the scale of reservoir groups expands, the difficulty of scheduling decisions also increases. This is primarily reflected in the continuous increase in decision variables, the increasing complexity of constraint conditions, and the diversification of scheduling objectives, posing more significant challenges to scheduling decisions^8^.

Various techniques have been developed over the past few decades to address reservoir scheduling problems^9^.These include linear programming^9–11^, nonlinear programming^12^ and dynamic programming^13–15^.However, each of these methods has its limitations. While nonlinear programming methods have their advantages, they tend to have relatively slow convergence speeds and require longer computation times, posing significant challenges in practical applications^16,17^. The dynamic programming approach, however, faces the so-called "curse of dimensionality," which refers to the exponential increase in computational complexity and resource requirements as the scale of the problem expands. This limitation restricts its application in large-scale reservoir scheduling problems^18,19^.To better address this issue, researchers have continuously explored optimization algorithms and achieved significant breakthroughs. Some nature-inspired optimization algorithms have been widely used in large-scale reservoir optimization models^20^, providing effective approaches for solving water resources system planning problems. He et al.^21^ proposed an Improved Sparrow Search Algorithm (ISSA) that integrates Cauchy mutation and reverse learning strategies to solve the joint scheduling problem of the Sanmenxia Reservoir and the Xiaolangdi Reservoir on the mainstream of the Yellow River; Wang et al.^22^ proposed a Yin-Yang Firefly Algorithm (YYFA) based on Cauchy mutation, which determines the initial position of fireflies through a Good Node Set (GNS) strategy to ensure the spatial representativeness of the firefly population; Cheng^23^ successfully applied the chaotic genetic algorithm to the scheduling of hydropower station reservoirs, demonstrating superior performance with significantly better convergence speed compared to dynamic programming and standard genetic algorithms; He et al.^24^ developed a slime mold algorithm combined with random reverse learning (HSMAAOA), which has also been applied to reservoir flood control scheduling, for the flood control scheduling of mixed reservoir groups. Hu Hexuan^25^ proposed a Q-learning algorithm combined with a penalty function. Although the penalty function method is often used in solving constrained problems, selecting penalty parameters is complex and difficult to grasp. If the penalty parameter is set too small, it will not effectively exert the role of the penalty function, and it will be difficult to fully restrict the constraint conditions. On the other hand, if the penalty parameter is set too large, it may introduce unnecessary calculation errors due to the accumulation of errors, affecting the accuracy of the solution. Although intelligent algorithms have been widely used in multiple fields and their powerful search capabilities are impressive, the issue of excessive randomness cannot be ignored. During the solution process, intelligent algorithms are prone to falling into local optimal solutions, leading to instability in the solution results, which may not be suitable for some application scenarios with high requirements for accuracy and stability^26^.

However, traditional optimization methods often appear inadequate when facing constrained and multi-constrained problems. Therefore, this paper proposes combining constraint-handling techniques in the solution process to deal more effectively with relevant constraints. Among them, the ε-constraint method, as a standard constraint handling method, has achieved remarkable results in constraint optimization problems in recent years. However, when facing high-dimensional issues, this method may encounter the challenge of the curse of dimensionality, resulting in insufficient guarantees for the convergence and global optimality of the algorithm. To overcome these limitations, this paper further explores other possible optimization strategies and techniques to achieve better results in solving the problem of reservoir flood control optimization scheduling. Literature^26^ proposes an innovative adaptive method based on the ε-constraint. This method prevents the algorithm from falling into the dilemma of locally optimal solutions by optimizing constraint handling techniques and adaptively setting ε values. This improvement not only enhances the search efficiency of the algorithm but also improves its robustness, demonstrating more robust performance in dealing with complex constraint conditions. The walrus optimization algorithm (WOA) is a new optimization algorithm proposed by Trojovsky et al. in 2023. Inspired by the natural behavior of walruses, the algorithm simulates a series of ecological behaviors such as feeding, migration, escaping, and defending against predators. WOA carefully designs three stages of exploration, migration, and development, achieving a balanced global search and local search capability. This allows the algorithm to extensively search potential solution spaces when solving optimization problems and conduct fine-grained searches and optimizations in local areas^27^. In literature^27^, simulation experiments compared the WOA algorithm with eight commonly used algorithms, including genetic algorithms, differential evolution algorithms, and gray wolf optimization algorithms. The results of the simulation experiments demonstrated that the WOA algorithm exhibited excellent convergence rates and solution accuracy in function optimization, significantly outperforming other similar algorithms, thus attracting widespread attention from the academic community. However, the WOA algorithm faces similar challenges to other optimization algorithms, namely limited local search capabilities and the tendency to get trapped in local optima, leading to the inability to find global optimal solutions. This paper proposes three key improvements to the WOA algorithm to overcome these limitations. Firstly, we integrate the SPM chaotic mapping to enrich the diversity of the initialized population, thereby improving the algorithm's search performance.

Secondly, by incorporating a spiral search strategy, we significantly enhance the algorithm's global optimization capabilities. Finally, combining Cauchy mutation and reverse learning strategies, we effectively address the issue of the algorithm easily getting trapped in local optima and accelerating convergence speed. To address multi-constraint and strong-constraint problems, this paper proposes an improved WOA algorithm coupled with adaptive ε-constraint (IWOA) and selects constraint optimization test functions for simulation experiments. The experimental results show that compared to methods such as ε-DE, ε-WOA and ε-PSO, the newly proposed algorithm obtains global optimal solutions and exhibits stronger robustness. To verify the effectiveness of the algorithm's practical applications, this paper takes the Taolinkou Reservoir, Daheiting Reservoir, and Panjiakou Reservoir in the upper and middle reaches of the Luanhe River as the research objects. It establishes a flood control scheduling model for the mixed reservoir group. The ε-IWOA algorithm is used for solving and compared with ε-DE, ε-WOA and ε-PSO algorithms through experiments. The research results indicate that the algorithm exhibits excellent optimization performance and possesses good robustness, making it a practical and effective method for solving reservoir flood control scheduling models.

The structure of this paper is as follows: section "The optimal flood control scheduling model for reservoir groups" elaborates on the construction process of the flood control optimization scheduling model for reservoir groups, providing a solid theoretical foundation for subsequent scheduling research. Section "Adaptive ε-constrained IWOA algorithm" introduces the ε-IWOA algorithm and verifies its performance and effectiveness in practical applications through a series of tests. The innovation and practicality of this algorithm provide technical solid support for subsequent case analysis. In Section "Case analysis", the case analysis section combines the joint scheduling of the reservoir group in the specific research area to deeply explore the practical application effects of the model and algorithm. It discusses and analyzes the results in detail. Finally, in Section "Conclusion", we summarize the main research results of the paper and draw corresponding conclusions.

## The optimal flood control scheduling model for reservoir groups

### Objective function

**Figure 1:**
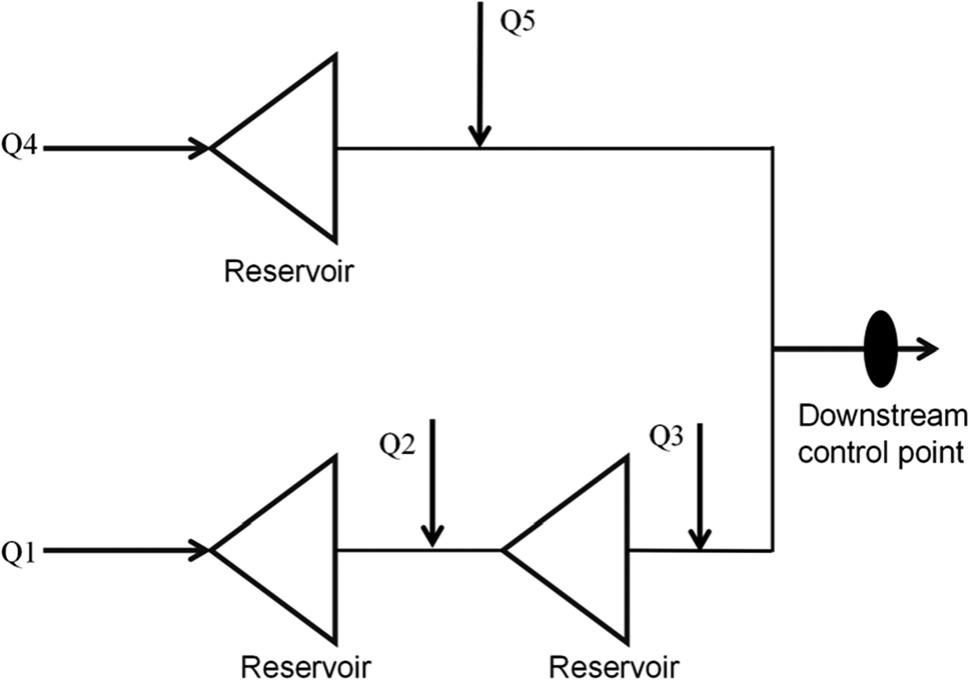
Generalized diagram of joint operation of reservoirs.

The flood control system for a river basin reservoir group typically encompasses multiple components such as reservoirs, dams, river channels, flood diversion areas, and downstream control points. Appreciatively large river basins may also include flood storage and detention areas. However, in the discussion of this paper, we do not delve into any content related to flood storage and detention areas. The generalized network structure of the flood control system can be visually presented in Fig. 1.

The objective of flood control scheduling for a reservoir group is to minimize the flood peak flow in the reservoirs and mitigate flood disasters in downstream protected areas. During the flood scheduling process, each reservoir's initial water level for flood control is set at the flood limit level. To prepare for the next flood event, the reservoir water level should be reduced to the flood limit level by the end of the current flood operation. This paper aims to control the flood volume in the reservoirs as much as possible during the scheduling process while minimizing the flood peak flow at the downstream control point. Taking a typical flood process as an example, the objective function of the optimization model is designed to achieve these objectives.

1 $$ ob = \min (\omega_{1} \cdot V_{1}^{\prime } + \omega_{2} \cdot V_{2}^{\prime } + \omega_{3} \cdot V_{3}^{\prime } + \omega_{4} \cdot V_{4}^{\prime } ) $$

where: $V_{1}^{\prime}$,$V_{2}^{\prime}$,$V_{3}^{\prime}$ represent the normalized maximum storage capacities of the three reservoirs after flood scheduling, in billions of m^3^ ;$Q^{\prime}$ represents the standardized peak flow at the downstream control point, m^3^/s; ω_1_, ω_2_, ω_3_, ω_4_are the weight factors for objectives 1, 2, 3, and 4, respectively (the weight factors in this paper are referenced from the research results of Chen et al.^28^).To eliminate the influence of different units, the original values can be normalized using Eqs. (2) and (3):

2 $$ V_{i}^{\prime} = \frac{{V_{Z,i} - V_{i} }}{{V_{z,i} - V_{l,i} }}(i = 1,2,3) $$

3 $$ Q^{\prime} = \frac{Q}{{Q_{\max } }} $$

In the formula: $V_{{\text{i}}}^{{}}$ is the maximum storage capacity of the i-th reservoir during flood control scheduling, in billions of m^3^;$V_{{\text{z,i}}}^{{}}$ is the total flood control capacity of the i-th reservoir, in billions of m^3^;$V_{l,i}$ is the storage capacity corresponding to the flood control limit water level of the i-th reservoir, in billions of m^3^; Q is the peak flow rate at the downstream control point during flood control scheduling, m^3^/s; Q_max_is the maximum discharge flow rate at the downstream control point without disaster based on historical data, m^3^/s; $V_{z,i} - V_{l,i}$ is the storage capacity between the flood control limit water level and the maximum water level of the i-th reservoir, in billions of m^3^, known as the maximum flood control storage capacity;$V_{i}^{\prime}$ is the proportion of flood storage occupied during the scheduling process of the i-th reservoir; for the i-th reservoir,$V_{i}^{,}$ the smaller the value of, the safer the reservoir is. Additionally, for the downstream control point,$Q^{\prime}$ the smaller the value, the safer the downstream area is.

### Constraints

(1) Water balance constraints

4 $$ V_{t + 1,n} - V_{t,n} = (Q_{t + 1,n} - q_{t + 1,n} ) \cdot \Delta t $$

where $V_{t + 1,n}$ is the capacity of the nth reservoir at the end of the period,10^8^ m^3^;$V_{t,n}$ is the initial nth reservoir capacity, 10^8^ m^3^;$Q_{t + 1,n}$ is the inflow to the nth reservoir in period t,m^3^/s;${\text{q}}_{t + 1,n}$ is the nth relief flow in period t,m^3^/s;$\Delta t$ is to calculate the step size.

(2) Water level constraints

5 $$ Z_{{{\text{min}},{\text{n}}}}^{{}} \le Z_{n}^{{}} \le Z_{{\max ,{\text{n}}}}^{{}} $$

where:$Z_{{\text{min,n}}}^{{}}$ , $Z_{{\text{max,n}}}^{{}}$, $Z_{{\text{n}}}^{{}}$ are the flood limit level of the nth reservoir, the flood defense high water level, and the water level during the flood control and scheduling process, respectively,m.

(3) Flood flow limitation levels

6 $$ {0} \le {\text{q}}_{{\text{n}}} \le q_{{\max ,{\text{n}}}} $$

where ${\text{q}}_{{\text{n}}}^{{}}$ is the flow from the nth reservoir;${\text{q}}_{{\text{max,n}}}^{{}}$ is the discharge capacity of the nth reservoir at each period, billion m^3^.

(4) Water level restrictions

7 $$ {\text{Z}}_{{{\text{end}},{\text{n}}}} = Z_{{e,{\text{n}}}} $$

where $Z_{{\text{end,n}}}^{{}}$, $Z_{{\text{e,n}}}^{{}}$ is the water level at the end of reservoir scheduling and the limit water level, respectively,m.

## Adaptive ε-constrained IWOA algorithm

### Improved walrus algorithm

#### Fusion of SPM chaotic mapping

The traditional whale optimization algorithm exhibits significant randomness during the initialization process, leading to a decline in the whale's foraging ability. Based on this, a method based on SPM chaotic mapping is proposed, which can effectively enhance the diversity and randomness of the population, thus improving the algorithm's optimization and convergence speed. As evidenced by literature^29^, SPM chaotic mapping demonstrates strong search capabilities and enriches the diversity of the population during initialization. The formula is as follows:

8 $$ x(t + 1) = \left\{ {\begin{array}{*{20}c} {\bmod (\frac{x(t)}{n} + u\sin (\pi x(t) + r,1),0 \le x(t) < n} \\ {\bmod (\frac{x(t)/n}{{0.5 - n}} + u\sin (\pi x(t)) + r,1),n \le x(t) < 0.5} \\ {\bmod (\frac{(1 - x(t))/n}{{0.5 - n}} + u\sin (\pi (1 - x(t))) + r,1),0.5 \le x(t) < 1 - n} \\ {\bmod (\frac{(1 - x(t))}{n} + u\sin (\pi (1 - x(t))) + r,1),1 - n \le x(t) < 1} \\ \end{array} } \right. $$

where n ∈ (0, 1), u ∈ (0, 1), the system is in a chaotic state, and r is a random number between 0 and 1.

#### Fusion spiral search strategy

Integrating the spiral search strategy^30^ enables the whales to possess multiple search paths to better adjust their positions, thus enhancing the global search performance of the algorithm. The updated formula for the whale's position with the spiral exploration is as follows:

9 $$ {\text{x}}_{id}^{t + 1} = \left\{ \begin{gathered} z \cdot x_{id}^{t} \cdot \exp ( - i/aT),R_{2} < ST \hfill \\ x_{id}^{t} + Q \cdot z,R_{2} \ge ST \hfill \\ z = \exp (b \times p) \cdot \cos (2\pi p) \hfill \\ \end{gathered} \right. $$

where z is the spiral exploration factor, b is the spiral shape constant, and p denotes the path coefficient, a random number.

#### Incorporating Cauchy variation and reverse learning strategies

Tizhoosh et al.^31^ proposed an innovative opposite learning strategy in 2005. The core idea of this strategy is to find the corresponding opposite solutions based on the currently existing solutions through the opposite learning method. Subsequently, the optimal solutions among these opposite solutions are evaluated and compared to select and retain the more excellent ones. This approach provides a new solution for optimization problems and helps to enhance the performance and efficiency of the algorithm. To further improve the whale optimization algorithm's ability to find optimal solutions, this paper introduced the concept of opposite learning. The mathematical representation is as follows:

10 $$ {\text{x}}_{{{\text{best}}}}^{\prime } (t) = ub + r \oplus (lb - x_{best} (t)) $$

11 $$ {\text{x}}_{{\text{i,j}}}^{t + 1} = x_{best}^{\prime } (t) + b_{1} \oplus (x_{best} (t) - x_{best}^{\prime } (t)) $$

where ${\text{x}}_{best}^{\prime } (t)$ is the reverse solution of the optimal solution for the t-th generation, Ub and lb are upper and lower bounds, respectively, and r is a 1 that follows the (0,1) standard uniform distribution × The random number matrix of d (d is the spatial dimension), b1 represents the information exchange control parameter, and the formula is as follows:

12 $$ {\text{b}}_{1} = (\frac{{T_{{{\text{max}}}} - t}}{{T_{\max } }})^{t} $$

By utilizing the Cauchy operator to update the objective, we can leverage its adjustment capabilities to avoid the algorithm getting trapped in local optima. The calculation formula is as follows:

13 $$ {\text{x}}_{new} (t + 1) = Cauchy \oplus x_{best} (t) $$

To further enhance the search capability of the optimization algorithm, this paper introduce a hybrid strategy that alternately executes the opposite learning strategy and the Cauchy operator perturbation strategy with a certain probability, thereby dynamically updating the target position. Through opposite learning, we can obtain opposite solutions, which greatly expands the search range of the algorithm and enhances its global search capability. Additionally, this paper set dynamically changing upper and lower bounds, ub and lb, respectively. Compared to strategies with fixed boundaries, this dynamic boundary approach is more conducive to the optimization process of the algorithm.

In the Cauchy mutation strategy, this paper utilize mutation operators to perform mutation operations on the current best position, generating new solutions. This strategy can effectively overcome the drawback of the algorithm easily getting trapped in local optima, thereby enhancing its local search capability. By alternately executing these two strategies, we can strike a balance between global and local searches, further improving the search efficiency and accuracy of the algorithm. The selection probability ps, which determines which strategy to choose for updating, is defined as follows:

14 $$ P{\text{s}} = - \exp \left( {1 - \frac{t}{{T_{\max } }}} \right)^{20} + \sigma $$

where is the adjustment factor, after many experiments, taking the value of 0.05 when the function optimization results are optimal.

#### Adaptive ε-constraint method

To efficiently explore and develop the search space while balancing population diversity and convergence, Takahama^32^ proposed an adaptive ε-constraint method.By adopting an improved individual comparison criterion, the information of excellent infeasible individuals is fully utilized to enhance the exploration capability of the search region and effectively avoid local optima issues. Additionally, the use of an adaptive ε-parameter adjustment strategy balances the relationship between feasible and infeasible individuals, strengthening the algorithm's search efficiency and robustness. These improvements help the algorithm solve optimization problems more comprehensively and efficiently, enabling it to find global optimal solutions^33^. The improvements are as follows:

(1) By optimizing the individual comparison criterion, having successfully enhanced the diversity of the population, ensuring that both feasible and infeasible regions evolve towards the global optimal solution. This improvement not only enriches the exploration range of the search space but also ensures the convergence of the algorithm, thereby improving the efficiency and accuracy of finding the global optimal solution. The criterion formula is as follows:

15 $$ x_{1} \;is\;better\;than\;x_{2} \Leftrightarrow \left\{ {\begin{array}{*{20}c} {f\left( {x_{1} } \right) < f\left( {x_{2} } \right),G(x_{1} ) = 0,G(x_{2} ) = 0} \\ {f\left( {x_{1} } \right) < f\left( {x_{2} } \right),0 < G(x_{1} ) \le \varepsilon ,0 < G(x_{2} ) \le \varepsilon } \\ {0 < G(x_{1} ) \le \varepsilon ,G(x_{2} ) > \varepsilon } \\ {G(x_{1} ) < G(x_{2} ),G(x_{1} ) > \varepsilon ,G(x_{2} ) > \varepsilon ,rand \le ps} \\ {f\left( {x_{1} } \right) < f\left( {x_{2} } \right),G(x_{1} ) > \varepsilon ,G(x_{2} ) > \varepsilon ,rand \le ps} \\ {f\left( {x_{1} } \right) < f\left( {x_{2} } \right),G(x_{1} ) = 0,0 < G(x_{2} ) \le \varepsilon } \\ {G(x_{1} ) = 0,G(x_{2} ) > \varepsilon } \\ {f\left( {x_{1} } \right) < f\left( {x_{2} } \right),0 < G(x_{1} ) \le \varepsilon ,G(x_{2} ) = 0} \\ \end{array} } \right. $$

where: G(x) is the magnitude of constraint violation, f(x) is the value of function fitness, and Ps is a random number on the interval [0.9, 1].

(2) When the feasible region is relatively narrow, the algorithm may generate a relatively large ε-value. This strategy aims to expand the search range, thereby increasing the possibility of the algorithm escaping from local optima. Conversely, when the feasible region is broader, the algorithm generates a smaller ε-value, which helps enhance the population's development capability and accelerate the convergence process. Based on this idea, this paper propose an adaptive ε-adjustment strategy that optimizes the algorithm's performance by dynamically adjusting the ε-value. The formula is as follows:

16 $$ \varepsilon \left( {\text{t}} \right) = \left\{ \begin{gathered} \varepsilon {(0)} \times e^{ - a \times (t/Te)} ,t \le T{\text{e}} \hfill \\ 0,t > Te \hfill \\ \end{gathered} \right. $$

17 $$ \varepsilon (0) = 0.6 \times \sum\limits_{{{\text{t}} = 1}}^{N} {G(X_{{\text{i}}} )} /N $$

18 $$ \alpha = \alpha_{\min } + \lambda \times (\alpha_{\max } - \alpha_{\min } ) $$

where Te is the number of truncated evolutionary iterations, N is the number of populations $\lambda$, and is the proportion of feasible individuals in the population. The value of Te is particularly important, as being too large may result in too many infeasible solutions during the iteration process, thereby affecting the convergence of the population. Being too small eliminates many infeasible individuals in the early stages of iteration, making it easy to fall into local optima.

### Steps of the ε-IWOA algorithm

**Figure 2:**
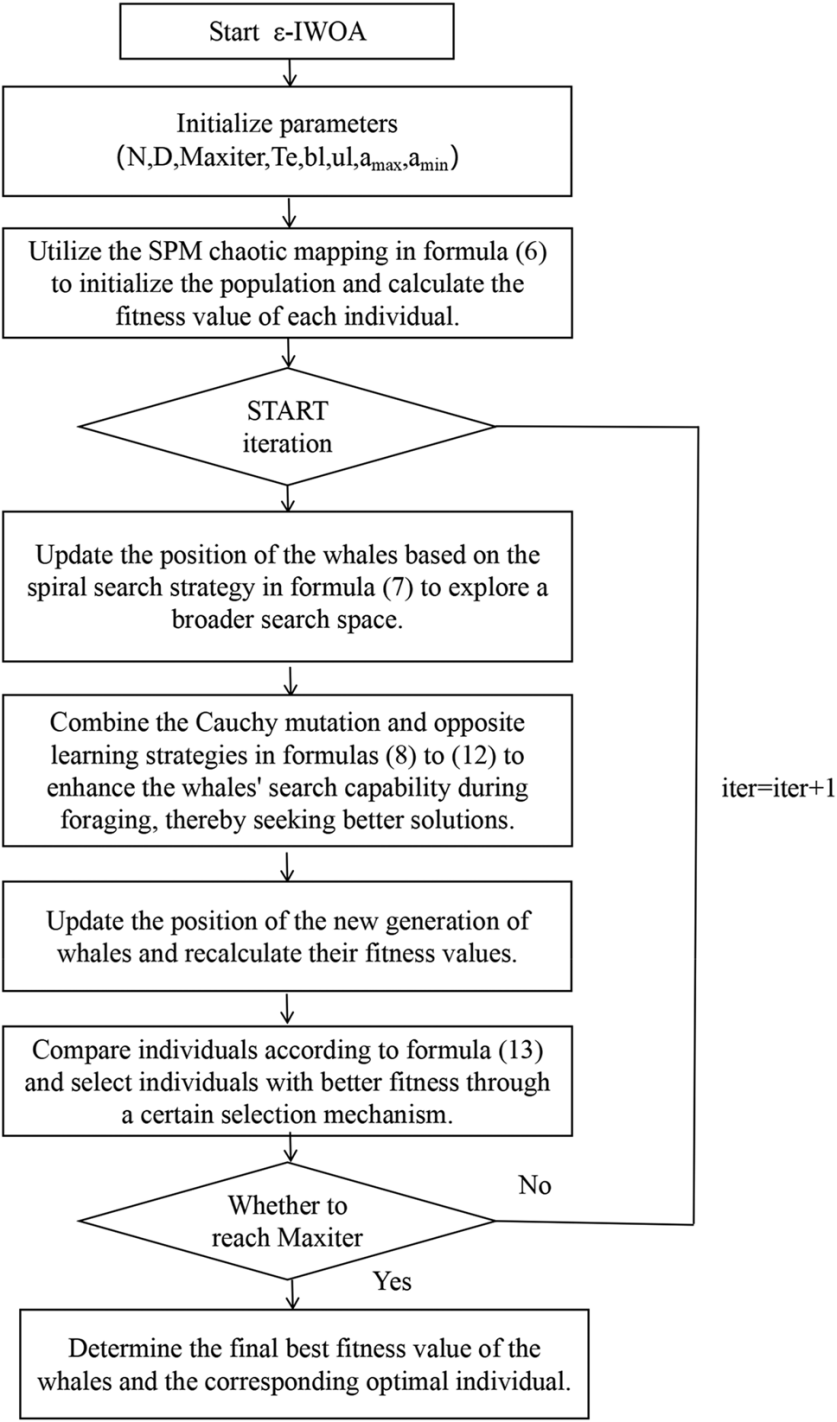
Flowchart of the ε-IWOA algorithm.

ε-IWOA algorithm flow with reference to Fig. 2 . The main steps are:

- Initialize parameters, including the maximum number of iterations T, population size N, boundary conditions (such as upper and lower bounds) ${\text{a}}_{\max }$,${\text{a}}_{\min }$ the dimension of decision variables, and the truncated evolution iteration count.
- Utilize the SPM chaotic mapping in formula (6) to initialize the population and calculate the fitness value of each individual.
- Update the position of the whales based on the spiral search strategy in formula (7) to explore a broader search space.
- Combine the Cauchy mutation and opposite learning strategies in formulas (8) to (12) to enhance the whales' search capability during foraging, thereby seeking better solutions.
- Update the position of the new generation of whales and recalculate their fitness values.
- Compare individuals according to formula (13) and select individuals with better fitness through a certain selection mechanism.
- Check if the termination criteria for iteration are met, such as reaching the maximum number of iterations or satisfying a convergence criterion. If the conditions are met, proceed to step h; otherwise, return to step c to continue iterating.
- Determine the final best fitness value of the whales and the corresponding optimal individual.

### ε-IWOA simulation experimental testing

To verify the effectiveness of the newly proposed ε-IWOA algorithm,this paper conducted a series of tests using 24 constrained optimization test functions. The results were then compared in detail with those obtained from the basic ε-WOA and ε-DE algorithms. In the experimental setup, this paper ensured that the population size for all three algorithms was set to 200, the maximum number of iterations was set to 20,000, the maximum number of function evaluations was limited to 500,000, the truncation evolution iteration number was set to 1000, and the tolerance for equation constraint violation was set to 0.0001.

To eliminate potential biases caused by the randomness of the algorithms, this paper ensured that each of the three algorithms was independently run 30 times for each test function. The experimental results showed that for each test problem, all three algorithms could find feasible solutions as the final optimal solutions in 30 independent runs. However, significant differences emerged in the quality of the optimal solutions obtained by different algorithms. To present this difference more visually, this paper compiled a statistical table (See Table 1 in the Appendix) showing the objective function values of the optimal solutions obtained by the three algorithms for a detailed comparative analysis, with bold font indicating the best performance.

The detailed data presented in the above table shows the significant effectiveness of the ε-constraint method in assisting the WOA algorithm in solving constrained optimization problems. The ε-IWOA algorithm achieved excellent optimization results in most tested functions by combining the improved WOA algorithm with the ε-constraint method. Notably, the ε-IWOA algorithm found the globally optimal solutions of the tasks and exhibited a minor standard deviation, indicating more stable results. Furthermore, the average values obtained by the ε-IWOA algorithm were lower than those of the other two comparison algorithms, demonstrating its excellent robustness.

For the more challenging functions g2 and g13, the ε-IWOA algorithm proposed in this paper exhibited significant advantages compared to the other two comparison algorithms. In terms of optimal values, the ε-IWOA algorithm found the optimal solutions successfully. It showed a minor standard deviation, fully demonstrating its superior performance in global search ability and optimization accuracy. On most of the tested functions, the ε-IWOA algorithm exhibited high stability in 30 solution experiments. Except for functions g20 and g22, the algorithm successfully found the optimal solutions that satisfied the constraint conditions.

This achievement was attributed to the introduction various innovative strategies in the basic WOA algorithm, including population initialization methods based on SPM chaotic mapping, spiral search strategies, and local intensification search strategies based on Cauchy mutation and reverse learning. The effective coupling of these strategies with the adaptive ε-constraint method jointly enhanced the optimization ability of the algorithm, achieving optimal levels in both solution accuracy and stability.

## Case analysis

### Overview of the research area

**Figure 3:**
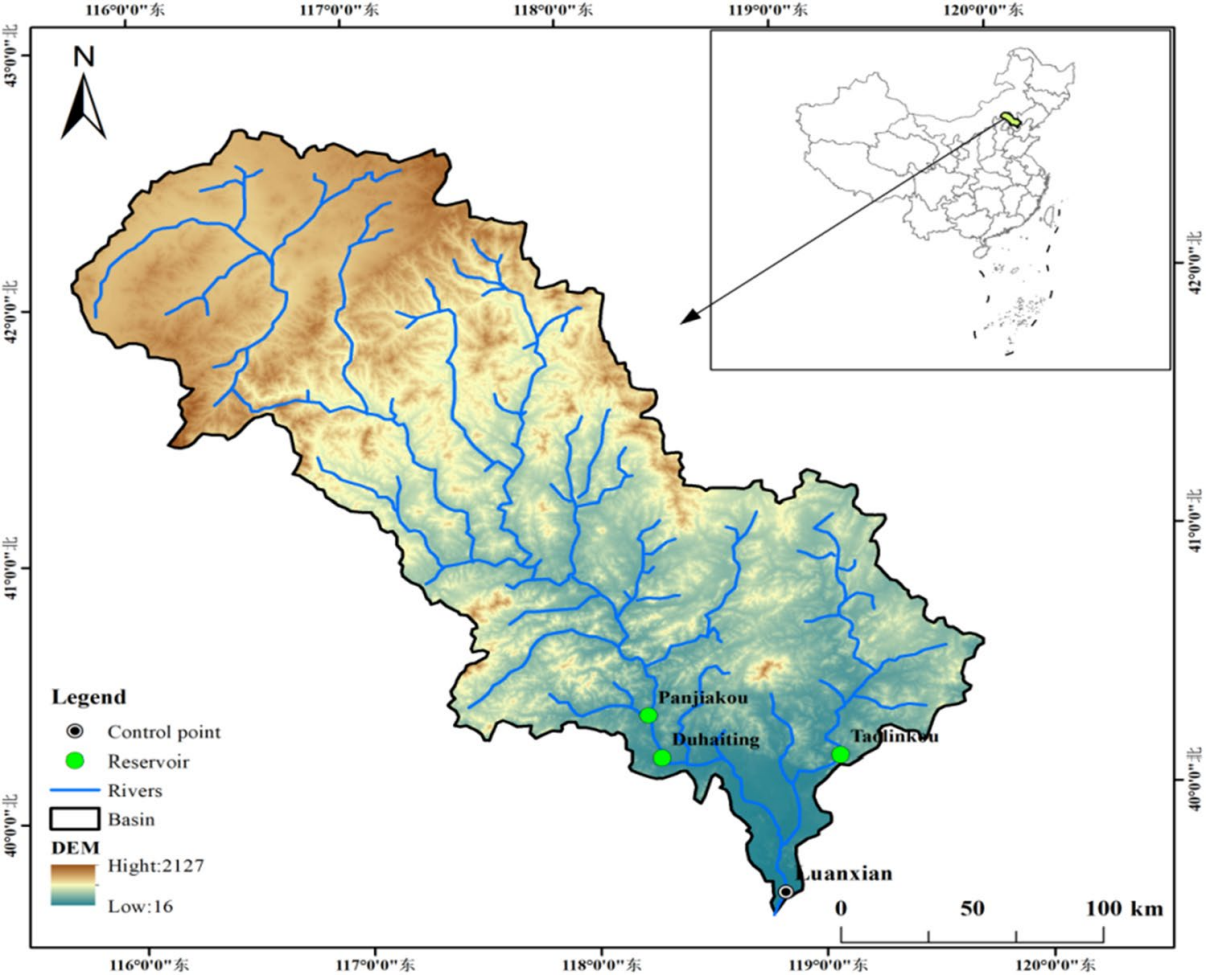
Water System Map of the Luanhe River Basin (Produced by ArcMap 10.8).

The Luanhe River Basin is located in the northeastern part of the North China Plain, with geographical coordinates spanning from 115° 34′ E to 119° 50′ E and from 39° 10′ N to 42° 30′ N. It boasts a vast drainage area of 44,800 km^2^. The mainstream of the Luanhe River runs southeast, traversing the Yanshan Mountains and the Jidong Plain, with a total length of 888 km. The climate in this region is characterized as a temperate continental monsoon climate with distinct seasonal features. The spring and autumn seasons are dry with little rainfall, while the winter is cold and arid. In contrast, the summer is hot and rainy. There is significant unevenness in precipitation distribution, with notable monthly variations. Specifically, summer rainfall ranges from 200 to 550 mm, accounting for 66% to 76% of the annual precipitation. July and August are the most rainy months, with their combined rainfall contributing 50% to 65% of the annual total. Due to the concentrated rainfall, the runoff of the Luanhe River fluctuates significantly throughout the year. Especially during the flood season in July and August, the inflow is particularly abundant, accounting for more than half of the annual total. Conversely, the inflow in January and February is relatively scarce, less than one-tenth of the annual total. Figure 3 depicts the water system map of the Luanhe River Basin, clearly illustrating the distribution and flow directions of the river system in this region.

In this study, we have selected three key reservoirs in the middle and lower reaches of the Luanhe River for in-depth investigation: the Taolinkou Reservoir, the Daheiting Reservoir, and the Panjiakou Reservoir. Table 1 provides a detailed overview of the key characteristics of these reservoirs. The Panjiakou Reservoir plays a pivotal role in the flood control and disaster reduction system of the Luanhe River. It not only can regulate floods but also effectively develops water resources and mitigates flood peaks when necessary, thereby reducing the impact of disasters. The Taolinkou Reservoir, located in the lower reaches of the Luanhe River near Tangshan, is a crucial agricultural water conservancy project. The high-quality surface water it provides is indispensable for soil improvement in the irrigation area downstream of the Luanhe River and for optimizing the growth environment of rice paddies. Meanwhile, the Daheiting Reservoir serves multiple functions, including coordinating with the Panjiakou Reservoir in water transfer and water level elevation, as well as trans-basin water transfer and retaining water from a wider area, providing strong support for regional water resource management.

**Table 1:** Parameters characterizing the reservoir.

Parameters	Panjiakou reservoir in Hebei province	Dahaiting reservoir	Touring the Taolinkou reservoir
Dead water level (m)	180.0	121.5	104.0
Flood limit level (m)	216.0	133.0	143.0
Design flood level (m)	224.5	133.0	143.4
Calibration flood level (m)	227.00	134.00	144.32
Maximum flood discharge flow (m^3^/s)	56,200	67,500	22,000
Flood protection storage capacity (10^8^m^3^)	29.30	3.37	8.59

### Flood evolution

The Xin'anjiang model, created by Zhao^34^, has been widely and successfully applied in the field of flood forecasting^35,36^. The core strategy of this model is to refine the complex watershed system into multiple sub-units. By meticulously calculating the runoff and confluence conditions of each sub-unit, the flooding process at the outlet of each sub-unit is accurately depicted. For detailed information on the theoretical background and parameter optimization of the Xin'anjiang model, please refer to Zhao's work^36^.

Taking Luanxian as a critical control point in the lower reaches of the Luanhe River, it effectively regulates all floods within this section. These floods are mainly triggered by high-intensity, long-duration, and widespread rainfall. The flooding process primarily consists of five key stages: floods upstream of the Panjiakou Reservoir, floods from Panjiakou to the Daheiting Reservoir, floods from Daheiting to Luanxian, floods upstream of the Taolinkou Reservoir, and floods from Taolinkou to Luanxian. Characterized by high flood peaks, short durations, and high flow velocities, these five types of floods often cause devastating disasters in downstream areas.

**Figure 4:**
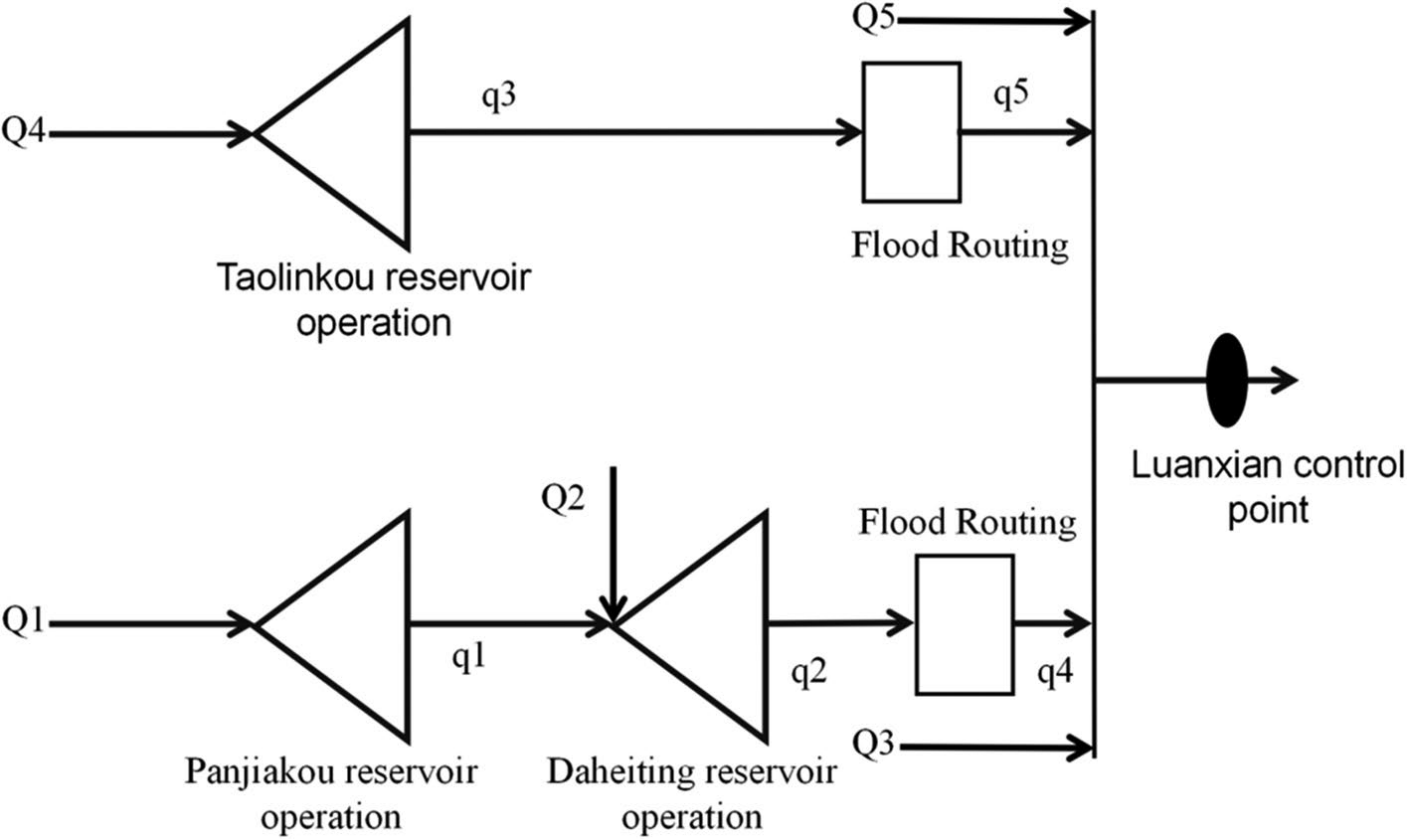
Evolution of Probable Floods in Luan River Basin.

During the flood evolution process in the Luanhe River Basin, as illustrated in Fig. 4 (Flood Process Generalized Diagram), this paper have several critical flood flows to consider. Assuming that the inflow to the Panjiakou Reservoir is Q1, the flood flow between Panjiakou and the Daheiting Reservoir is Q2, the flood flow between Daheiting and the control point of Luanxian is Q3, the flood flow upstream of the Taolinkou Reservoir is Q4, and the flood flow between Taolinkou and Luanxian is Q5. After regulation by the Panjiakou Reservoir, its outflow becomes q1. The inflow to the Daheiting Reservoir is the sum of the flow Q2 from the Panjiakou to Daheiting interval and the outflow q1 from the Panjiakou Reservoir. After regulation by the Daheiting Reservoir, its outflow becomes q2, which transforms into the flow q4 after the flood evolution process. Similarly, the outflow from the Taolinkou Reservoir is q3, which transforms into the flow q5 after flood evolution. This process reveals the complex dynamics of flood generation and evolution in the Luanhe River Basin.

**Figure 5:**
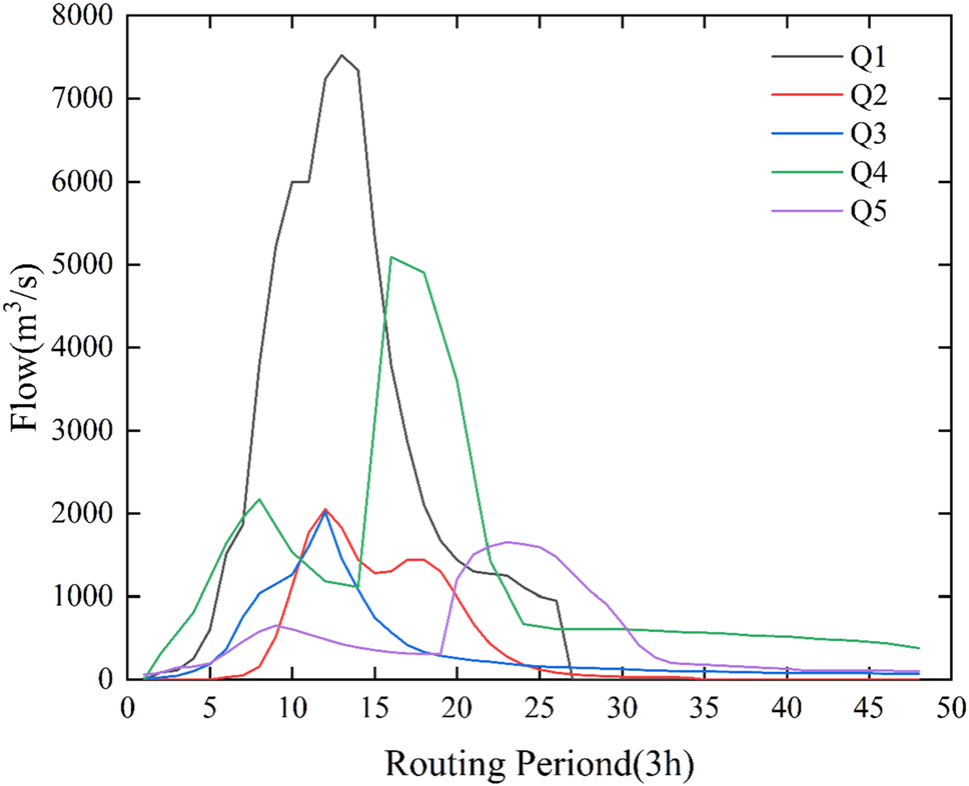
Flood forecast for five zones during a typical rainfall event.

In this study, the Xin'anjiang model was utilized to conduct a thorough and detailed flood process forecast for the five regions previously delineated. Figure 5 specifically illustrates the rainfall patterns in these five sub-regions during a typical flood event. This flood forecast spans 144 h, with precise analysis conducted at a three-hour calculation cycle.

The downstream water flows from Daheiting Reservoir, and Taolinkou Reservoir continues to move towards Luan County along the river. Regarding the process of flood evolution, this paper have adopted the linear Muskingum flood routing method for calculation, with specific details as follows:

19 $$ S(t) = k\left[ {{\text{x}}I(t) + (1 - x)O(t)} \right] $$

In the Muskingum flood routing model, S(t) represents the storage capacity of the river at time t; K represents the storage time constant of the river segment, and x represents the weight factor. I(t) and O(t) denote the inflow and outflow rates at time t, respectively. The parameters of the Muskingum model for the study area have been determined. The combined process of the three reservoirs is described as follows:

The inflow rate to Panjiakou Reservoir is denoted as Q1, and its outflow rate is q1. The inflow rate to Daheiting Reservoir consists of two parts: the local inflow rate Q2 and the outflow rate q1 from Panjiakou Reservoir. Since Panjiankou Reservoir is relatively close to Daheiting Reservoir, q1 does not require additional adjustment calculations and is directly considered a component of the inflow rate to Daheiting Reservoir. The outflow rate from Daheiting Reservoir is q2.

The inflow rate to Taolinkou Reservoir is Q4, and its outflow rate is q3.

At the Luan County control point, the flow rate Q consists of the following four components: the outflow rate q4 from Daheiting Reservoir, the outflow rate q5 from Taolinkou Reservoir, the inflow rate Q3 between Daheiting Reservoir and the Luan County control point, and the inflow rate Q5 between Taolinkou Reservoir and the Luan County control point.

### Results and discussion

#### Analysis of scheduling results

**Figure 6:**
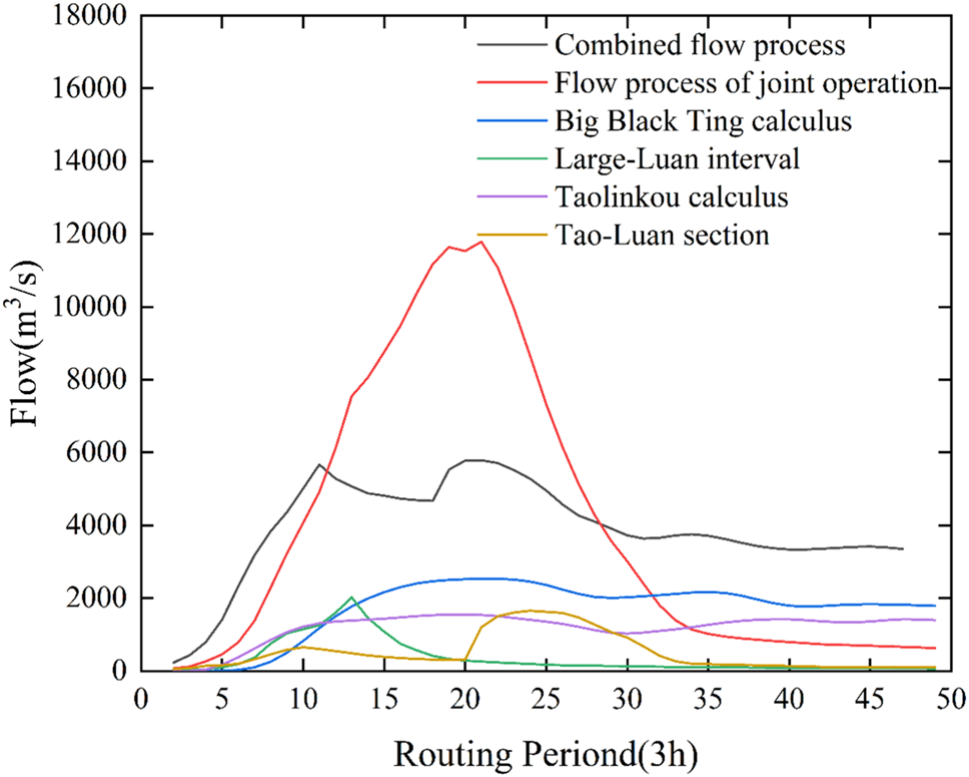
Flooding process in Luanxian.

The core of reservoir flood control scheduling lies in precisely implementing flood discharge, flood detention, and flood storage operations. Through these scientific measures, we can effectively reduce the property losses caused by flood disasters and promote the stable and efficient development of reservoir scheduling work. To further optimize the joint scheduling strategy for the reservoir group of Taolinkou Reservoir, Daheiting Reservoir, and Panjiakou Reservoir in the Luanhe River Basin, we introduced the ε-IWOA algorithm. In the algorithm parameter settings, we set the population size POP to 200, the maximum number of iterations T to 200,000, and the truncation iteration count Te to 1000. To ensure the accuracy of the solution, the paper conducted 20 independent operations, resulting in the optimal joint flood control optimization scheduling scheme, as detailed in Table 2. See Figs.1–3 in the Appendix visually present these three reservoirs' flood control scheduling details. The optimized joint scheduling scheme after the ε-IWOA algorithm adjustment shows the original combined flow process, the flood regulation discharge of each reservoir, the combined flow process, and the incoming water situation at the Luanxian control point, as illustrated in Fig. 6. From the chart data, it is evident that the peak clipping rates of Taolinkou Reservoir, Daheiting Reservoir, and Panjiakou Reservoir reached 65.41%, 30.07%, and 69.17%, respectively, while the peak clipping rate at the Luanxian control point was as high as 49.00%.

**Table 2:** Statistics of optimized scheduling results for joint flood control.

Reservoir downstream control point	Starting water level /m	Maximum occupied flood control capacity /10^8^ m^3^	Maximum flood peak /m^3^/s	Percentage of flood control capacity occupied/%	Percentage of peak reduction/%
Luan County	/	/	6009.96	/	49.00
Tao Lin Kou	138.5	1.42	1760.46	89.32	65.41
Daheiting	127.0	1.112	2806.26	90.02	30.07
Panjiakou	216.0	4.195	2318.49	80.95	69.17

This case analysis demonstrates the efficiency and practicality of the ε-IWOA algorithm in flood control scheduling for reservoir groups and showcases its unique advantages in solving similar problems. The ε-IWOA constrained algorithm provides powerful technical support for reservoir scheduling work, enabling us to carry out more scientific and precise flood control scheduling for reservoirs and ensure reservoir operations' safety and efficiency.

#### Comparison of scheduling results

To further validate the superior performance of the ε-IWOA algorithm in reservoir flood control scheduling, this paper conducted comparative tests using ε-WOA, ε-DE, and ε-PSO algorithms under strictly identical conditions to solve the model. Regrettably, after multiple attempts, none of these three algorithms could find a feasible solution for the model. This result further highlights the significant advantage of the ε-IWOA algorithm in solving reservoir scheduling problems. See Figs. 4–6 in the Appendix (only showing the scheduling results for the Panjiakou Reservoir) visually present the solution results of ε-WOA, ε-DE, and ε-PSO algorithms, which are inferior to the ε-IWOA algorithm in terms of performance. These comparative experiments demonstrate the effectiveness of the ε-IWOA algorithm and provide strong support for its application in practical reservoir scheduling.

## Conclusion

Based on the fundamental whale optimization algorithm, this paper innovatively proposes the ε-IWOA algorithm by incorporating the population initialization method based on SPM chaotic mapping, spiral search strategy, and combining Cauchy mutation with reverse learning techniques. After conducting thorough research, the following main conclusions are drawn:

1. For the joint scheduling problem of Taolinkou Reservoir, Daheiting Reservoir, and Panjiakou Reservoir in the Luanhe River Basin, this paper proposes an adaptive ε-IWOA algorithm. It compares it with ε-DE, ε-WOA, and ε-PSO algorithms. The experimental results indicate that ε-IWOA algorithm performs the best in optimization, with the occupied flood control capacity of the three reservoirs reaching 89.32%, 90.02%, and 80.95%, respectively. The control points in Luan County can reduce the peak by 49%. This fully validates the efficiency and practicality of the ε-IWOA algorithm in flood control optimization scheduling problems for reservoir groups, providing a new, practical approach to solving reservoir scheduling models.
2. To comprehensively evaluate the performance of the ε-IWOA algorithm, This paper selected 24 constrained test functions for simulation experiments and compared them with ε-DE, ε-WOA and ε-PSO algorithms. The experimental results show that the ε-IWOA algorithm significantly outperforms computation speed and solution accuracy, especially when dealing with high-dimensional, nonlinear, and strongly constrained combination problems. This further verifies the superiority and effectiveness of the ε-IWOA algorithm in solving complex constrained optimization problems.
3. Looking ahead, we will continue exploring other methods to improve and optimize the ε-IWOA algorithm to meet the more complex needs of joint scheduling for reservoir groups. Meanwhile, this efficient optimization algorithm also has the potential to be extended to other engineering-constrained optimization problems, providing powerful technical support for solving practical engineering issues.

### Supplementary Information

Supplementary Information.

## Data Availability

The datasets generated and/or analyzed during the current study are not publicly available but are available from the corresponding author on reasonable request.
