# Supplementary material for: Research on hybrid reservoir scheduling optimization based on improved walrus optimization algorithm with coupling adaptive ε constraint and multi-strategy optimization
Source: Sci Rep. 2024 May 25;14:11981. doi: 10.1038/s41598-024-62722-8 (PMC11128023; doi:10.1038/s41598-024-62722-8)
Supplement: Supplementary file 1 — Supplementary Information. [file 41598_2024_62722_MOESM1_ESM.pdf]

# Research on Hybrid Reservoir Scheduling Optimization Based on Improved Walrus Optimization Algorithm with Coupling Adaptive ε Constraint and Multi-strategy Optimization

Ji He[1],Yefeng Tang[1],Xiaoqi Guo[1],Haitao Chen[1*],Wen Guo[1]

1. College of Water Resources, Henan Key Laboratory of Water Resources Conservation and Intens ive Utilization in the Yellow River Basin, North China University of Water Resources and Electric Power, Zhengzhou 450046, China; heji15005@163.com(Ji He);15850352835@163.com(Yefeng Tan g);guoxiaoqi417@163.com(Xiaoqi Guo);zzchenhaitao@126.com(Haitao Chen);gw681014@163.com(We n Guo).

*Correspondence:zzchenhaitao@126.com(Haitao Chen)

Table 1. Comparison of Results Obtained by ε-IWOA, ε-WOA, ε-DE, and ε-PSO on 24 Constrained Optimization Test Functions

| Function | Theoretical Optimality | Arithmetic | Best | Wost | Average value | SD |
|---|---|---|---|---|---|---|
| g01 | -15.000 | **ε-DE** | **$-1.5\times10^{1}$** | **$-1.5\times10^{1}$** | **$-1.5\times10^{1}$** | **0** |
| | | ε-WOA | $-1.26562\times10^{1}$ | $-1.5\times10^{1}$ | $-1.35889\times10^{1}$ | $1.1568\times10^{0}$ |
| | | **ε-PSO** | **$-1.5\times10^{1}$** | **$-1.5\times10^{1}$** | **$-1.5\times10^{1}$** | **0** |
| | | **ε-IWOA** | **$-1.5\times10^{1}$** | **$-1.5\times10^{1}$** | **$-1.5\times10^{1}$** | **0** |
| g02 | -0.803619 | ε-DE | $-6.592\times10^{-1}$ | $-2.944\times10^{-1}$ | $-4.5337\times10^{-1}$ | $1.61\times10^{-1}$ |
| | | ε-WOA | $-6.466\times10^{-1}$ | $-7.9131\times10^{-1}$ | $-7.207\times10^{-1}$ | $3.53\times10^{-2}$ |
| | | ε-PSO | $-6.804\times10^{-1}$ | $-76.71\times10^{-1}$ | $-7.404\times10^{-1}$ | $4.34\times10^{-2}$ |
| | | **ε-IWOA** | **$-7.9856\times10^{-1}$** | **$-8.0360\times10^{-1}$** | **$-8.0318\times10^{-1}$** | **$9.0482\times10^{-3}$** |
| g03 | -1.000 | ε-DE | $-4.8507\times10^{-1}$ | $-7.84581\times10^{-3}$ | $-1.85324\times10^{-1}$ | $1.2768\times10^{-1}$ |
| | | ε-WOA | $-1.0005\times10^{0}$ | $-1.0005\times10^{0}$ | -1.0005 | $2.6451\times10^{-5}$ |
| | | ε-PSO | $-1.0005\times10^{0}$ | $-1.0005\times10^{0}$ | -1.0005 | $2.5284\times10^{-5}$ |
| | | **ε-IWOA** | **$-1.0005\times10^{0}$** | **$-1.0005\times10^{0}$** | **$-1.0005\times10^{0}$** | **$2.7545\times10^{-6}$** |

| | | | | | | |
|---|---|---|---|---|---|---|
| | | ε-DE | **$-3.0666\times10^{4}$** | **$-3.0666\times10^{4}$** | **$-3.0666\times10^{4}$** | **$1.361\times10^{-11}$** |
| | | ε-WOA | **$-3.0666\times10^{4}$** | **$-3.0666\times10^{4}$** | **$-3.0666\times10^{4}$** | **$1.361\times10^{-11}$** |
| g04 | -30665.539 | ε-PSO | **$-3.0666\times10^{4}$** | **$-3.0666\times10^{4}$** | **$-3.0666\times10^{4}$** | **$1.361\times10^{-11}$** |
| | | **ε-IWOA** | **$-3.0666\times10^{4}$** | **$-3.0666\times10^{4}$** | **$-3.0666\times10^{4}$** | **$1.361\times10^{-11}$** |
| | | ε-DE | $5.1265\times10^{3}$ | $6.0487\times10^{3}$ | $5.3561\times10^{3}$ | $2.8534\times10^{2}$ |
| | | ε-WOA | 5136.1016 | 5126.4967 | 5126.4758 | $2.245\times10^{2}$ |
| g05 | 5126.4967 | ε-PSO | $5.1378\times10^{3}$ | 5.126.4967 | $5.3561\times10^{3}$ | $2.578\times10^{2}$ |
| | | **ε-IWOA** | **$5.1265\times10^{3}$** | **$5.1265\times10^{3}$** | **$5.1265\times10^{3}$** | **$9.5896\times10^{-13}$** |
| | | ε-DE | **$-6.9618\times10^{3}$** | **$-6.9618\times10^{3}$** | **$-6.9618\times10^{3}$** | **$1.84\times10^{-12}$** |
| | | ε-WOA | **$-6.9618\times10^{3}$** | **$-6.9618\times10^{3}$** | **$-6.9618\times10^{3}$** | **$1.84\times10^{-12}$** |
| g06 | -6961.8139 | ε-PSO | **$-6.9618\times10^{3}$** | **$-6.9618\times10^{3}$** | **$-6.9618\times10^{3}$** | **$1.84\times10^{-12}$** |
| | | **ε-IWOA** | **$-6.9618\times10^{3}$** | **$-6.9618\times10^{3}$** | **$-6.9618\times10^{3}$** | **$1.84\times10^{-12}$** |
| | | ε-DE | $2.4400\times10^{1}$ | $2.4485\times10^{1}$ | $2.4435\times10^{1}$ | $6.3\times10^{-6}$ |
| | | ε-WOA | $2.43062\times10^{1}$ | $2.43062\times10^{1}$ | $2.43062\times10^{1}$ | $1.5674\times10^{-6}$ |
| g07 | 24.3062 | ε-PSO | $2.4215\times10^{1}$ | $2.43062\times10^{1}$ | $2.43062\times10^{1}$ | $2.1357\times10^{-6}$ |
| | | **ε-IWOA** | **$2.43062\times10^{1}$** | **$2.43062\times10^{1}$** | **$2.43062\times10^{1}$** | **$1.3004\times10^{-6}$** |
| | | ε-DE | $-9.5800\times10^{-2}$ | $-2.7263\times10^{-2}$ | $-9.1254\times10^{-2}$ | $1.902\times10^{-2}$ |
| | | ε-WOA | $-9.5800\times10^{-2}$ | $-9.5800\times10^{-2}$ | $-9.5800\times10^{-2}$ | $1.9216\times10^{-17}$ |
| g08 | -0.0958 | ε-PSO | $-9.5800\times10^{-2}$ | $-5.245\times10^{-2}$ | $-9.521\times10^{-2}$ | $1.854\times10^{-8}$ |
| | | **ε-IWOA** | **$-9.5800\times10^{-2}$** | **$-9.5800\times10^{-2}$** | **$-9.5800\times10^{-2}$** | **$1.8916\times10^{-17}$** |
| | | ε-DE | $6.8063\times10^{2}$ | $6.8063\times10^{2}$ | $6.8063\times10^{2}$ | $1.9027\times10^{-13}$ |
| g09 | 680.6300 | ε-WOA | $6.8063\times10^{2}$ | $6.8063\times10^{2}$ | $6.8063\times10^{2}$ | $5.9687\times10^{-13}$ |
| | | ε-PSO | $6.8063\times10^{2}$ | $6.8063\times10^{2}$ | $6.8063\times10^{2}$ | $4.203\times10^{-13}$ |
| | | **ε-IWOA** | **$6.8063\times10^{2}$** | **$6.8063\times10^{2}$** | **$6.8063\times10^{2}$** | **$3.59864\times10^{-13}$** |

| | | | | | | |
|---|---|---|---|---|---|---|
| | | ε-DE | $7.1198\times10^{3}$ | $7.4690\times10^{3}$ | $7.2236\times10^{2}$ | $5.578\times10^{-8}$ |
| g10 | 7049.2480 | ε-WOA | $7.0492\times10^{3}$ | $7.0492\times10^{3}$ | $7.0492\times10^{3}$ | $6.4512\times10^{-8}$ |
| | | ε-PSO | $7.0492\times10^{3}$ | $7.0492\times10^{3}$ | $7.0492\times10^{3}$ | $6.2123\times10^{-8}$ |
| | | **ε-IWOA** | **$7.049248\times10^{3}$** | **$7.049248\times10^{3}$** | **$7.049248\times10^{3}$** | **$2.6195\times10^{-12}$** |
| | | ε-DE | $7.4990\times10^{-1}$ | $7.5867\times10^{-1}$ | $7.5027\times10^{-1}$ | $1.6789\times10^{12}$ |
| g11 | 0.7499 | ε-WOA | $7.4990\times10^{-1}$ | $7.4990\times10^{-1}$ | $7.4990\times10^{-1}$ | $1.5161\times10^{-12}$ |
| | | ε-PSO | $7.4990\times10^{-1}$ | $7.5378\times10^{-1}$ | $7.4990\times10^{-1}$ | $1.5582\times10^{-12}$ |
| | | **ε-IWOA** | **$7.4990\times10^{-1}$** | **$7.4990\times10^{-1}$** | **$7.4990\times10^{-1}$** | **$1.2156\times10^{-12}$** |
| | | ε-DE | **$-1.000\times10^{0}$** | **$-1.000\times10^{0}$** | **$-1.000\times10^{0}$** | **0** |
| g12 | -1.0000 | ε-WOA | **$-1.000\times10^{0}$** | **$-1.000\times10^{0}$** | **$-1.000\times10^{0}$** | **0** |
| | | ε-PSO | **$-1.000\times10^{0}$** | **$-1.000\times10^{0}$** | **$-1.000\times10^{0}$** | **0** |
| | | **ε-IWOA** | **$-1.000\times10^{0}$** | **$-1.000\times10^{0}$** | **$-1.000\times10^{0}$** | **0** |
| | | ε-DE | $4.8479\times10^{-2}$ | $3.9856\times10^{-2}$ | $1.4576\times10^{-2}$ | $2.2367\times10^{-1}$ |
| g13 | 0.0539 | ε-WOA | $4.3887\times10^{-2}$ | $5.3925\times10^{-2}$ | $6.9634\times10^{-2}$ | $7.6786\times10^{-2}$ |
| | | ε-PSO | $4.5671\times10^{-2}$ | $4.2553\times10^{-2}$ | $6.7124\times10^{-2}$ | $6.4246\times10^{-2}$ |
| | | **ε-IWOA** | **$5.14\times10^{-2}$** | **$5.39\times10^{-2}$** | **$5.3543\times10^{-2}$** | **$9.3686\times10^{-4}$** |
| | | ε-DE | $-4.6101\times10^{1}$ | $-3.6886\times10^{1}$ | $-4.8893\times10^{1}$ | 2.47896e$\times10^{-1}$ |
| g14 | -47.7649 | ε-WOA | $-4.7765\times10^{1}$ | $-4.7765\times10^{1}$ | $-4.7765\times10^{1}$ | $3.5897\times10^{-1}$ |
| | | ε-PSO | $-4.7765\times10^{1}$ | $-4.7765\times10^{1}$ | $-4.7765\times10^{1}$ | $3.4257\times10^{-1}$ |
| | | **ε-IWOA** | **$-4.7765\times10^{1}$** | **$-4.7765\times10^{1}$** | **$-4.7765\times10^{1}$** | **$3.1568\times10^{-4}$** |
| | | ε-DE | $9.6172\times10^{2}$ | $9.6690\times10^{2}$ | $9.6389\times10^{2}$ | $2.6101\times10^{-13}$ |
| g15 | 961.7150 | ε-WOA | $9.6172\times10^{2}$ | $9.6690\times10^{2}$ | $9.6389\times10^{2}$ | $6.894\times10^{-13}$ |
| | | ε-PSO | $9.6172\times10^{2}$ | $9.6690\times10^{2}$ | $9.6389\times10^{2}$ | $4.2578\times10^{-13}$ |

| | | | | | | |
|---|---|---|---|---|---|---|
| | | **ε-IWOA** | $9.6172\times10^{2}$ | $9.6690\times10^{2}$ | $9.6389\times10^{2}$ | **$5.489\times10^{-14}$** |
| | | ε-DE | $-1.9052\times10^{0}$ | $-1.9052\times10^{0}$ | $-1.9052\times10^{0}$ | $8.5952\times10^{-14}$ |
| g16 | -1.9052 | ε-WOA | $-1.9052\times10^{0}$ | $-1.9052\times10^{0}$ | $-1.9052\times10^{0}$ | $6.9887\times10^{-16}$ |
| | | ε-PSO | $-1.9052\times10^{0}$ | $-1.9052\times10^{0}$ | $-1.9052\times10^{0}$ | $7.6811\times10^{-15}$ |
| | | **ε-IWOA** | **$-1.9052\times10^{0}$** | **$-1.9052\times10^{0}$** | **$-1.9052\times10^{0}$** | **$2.751\times10^{-16}$** |
| | | ε-DE | $8.8755\times10^{3}$ | $9.7762\times10^{3}$ | $9.1301\times10^{3}$ | $1.1178\times10^{2}$ |
| g17 | 8853.5339 | ε-WOA | $8.8108\times10^{3}$ | $8.9457\times10^{3}$ | $8.9452\times10^{3}$ | $4.8569\times10^{1}$ |
| | | ε-PSO | $8.8543\times10^{3}$ | $9.2377\times10^{3}$ | $8.9864\times10^{3}$ | $2.3821\times10^{2}$ |
| | | **ε-IWOA** | **8.8535**$\times10^{3}$ | **8.9563**$\times10^{3}$ | **8.8756**$\times10^{3}$ | **3.6678**$\times10^{1}$ |
| | | ε-DE | $-8.6558\times10^{-1}$ | $-8.6267\times10^{-1}$ | $-8.6409\times10^{-1}$ | $6.9167\times10^{-4}$ |
| g18 | -0.8660 | ε-WOA | $-8.6600\times10^{-1}$ | $-8.6600\times10^{-1}$ | $-8.6600\times10^{-1}$ | $5.8956\times10^{-4}$ |
| | | ε-PSO | $-8.6448\times10^{-1}$ | $-8.6327\times10^{-1}$ | $-8.6527\times10^{-1}$ | $7.8537\times10^{-4}$ |
| | | **ε-IWOA** | **$-8.6600\times10^{-1}$** | **$-8.6600\times10^{-1}$** | **$-8.6600\times10^{-1}$** | **$1.3598\times10^{-8}$** |
| | | ε-DE | $3.4589\times10^{1}$ | $3.6689\times10^{1}$ | $3.4467\times10^{1}$ | $5.1896\times10^{-1}$ |
| g19 | 32.6556 | ε-WOA | $3.2655\times10^{1}$ | $3.26576\times10^{1}$ | $3.2656\times10^{1}$ | $4.442\times10^{-1}$ |
| | | ε-PSO | $3.5822\times10^{1}$ | $3.6582\times10^{1}$ | $3.2332\times10^{1}$ | $4.879\times10^{-1}$ |
| | | **ε-IWOA** | **$3.2656\times10^{1}$** | **$3.2656\times10^{1}$** | **$3.2656\times10^{1}$** | **$3.6859\times10^{-2}$** |
| | | ε-DE | — | — | — | — |
| g20 | 0.2049 | ε-WOA | — | — | — | — |
| | | ε-PSO | — | — | — | — |
| | | **ε-IWOA** | — | — | — | — |
| | | ε-DE | $1.9372\times10^{2}$ | $1.000\times10^{2}$ | $5.2372\times10^{2}$ | $8.6672\times10^{2}$ |
| g21 | 193.7245 | ε-WOA | $8.4856\times10^{2}$ | $3.2396\times10^{3}$ | $2.5548\times10^{2}$ | $5.2789\times10^{1}$ |
| | | ε-PSO | $1.9372\times10^{2}$ | $1.854\times10^{2}$ | $2.542\times10^{2}$ | $3.2145\times10^{1}$ |

| | | | | | | |
|---|---|---|---|---|---|---|
| | | **ε-IWOA** | **$1.9372\times10^{2}$** | **$1.9372\times10^{2}$** | **$1.9372\times10^{2}$** | **$8.6577\times10^{-12}$** |
| | | ε-DE | — | — | — | — |
| g22 | 236.4310 | ε-WOA | — | — | — | — |
| | | ε-PSO | — | — | — | — |
| | | **ε-IWOA** | — | — | — | — |
| | | ε-DE | $-1.687\times10^{2}$ | $9.000\times10^{2}$ | $4.8654\times10^{2}$ | $4.4465\times10^{2}$ |
| g23 | -400.0551 | ε-WOA | $-5.3007\times10^{2}$ | $-4.0006\times10^{2}$ | $-3.3564\times10^{-1}$ | $2.7458\times10^{2}$ |
| | | ε-PSO | $-2.478\times10^{2}$ | $-8.512\times10^{2}$ | $-4.257\times10^{-1}$ | $3.5245\times10^{2}$ |
| | | **ε-IWOA** | **$-4.0006\times10^{2}$** | **$-4.0006\times10^{2}$** | **$-4.0006\times10^{2}$** | **$2.2005\times10^{-4}$** |
| | | ε-DE | **$-5.5080\times10^{0}$** | **$-5.5080\times10^{0}$** | **$-5.5080\times10^{0}$** | **$1.8067\times10^{-15}$** |
| g24 | -5.5080 | ε-WOA | **$-5.5080\times10^{0}$** | **$-5.5080\times10^{0}$** | **$-5.5080\times10^{0}$** | **$1.8067\times10^{-15}$** |
| | | ε-PSO | **$-5.5080\times10^{0}$** | **$-5.5080\times10^{0}$** | **$-5.5080\times10^{0}$** | **$1.8067\times10^{-15}$** |
| | | **ε-IWOA** | **$-5.5080\times10^{0}$** | **$-5.5080\times10^{0}$** | **$-5.5080\times10^{0}$** | **$1.8067\times10^{-15}$** |

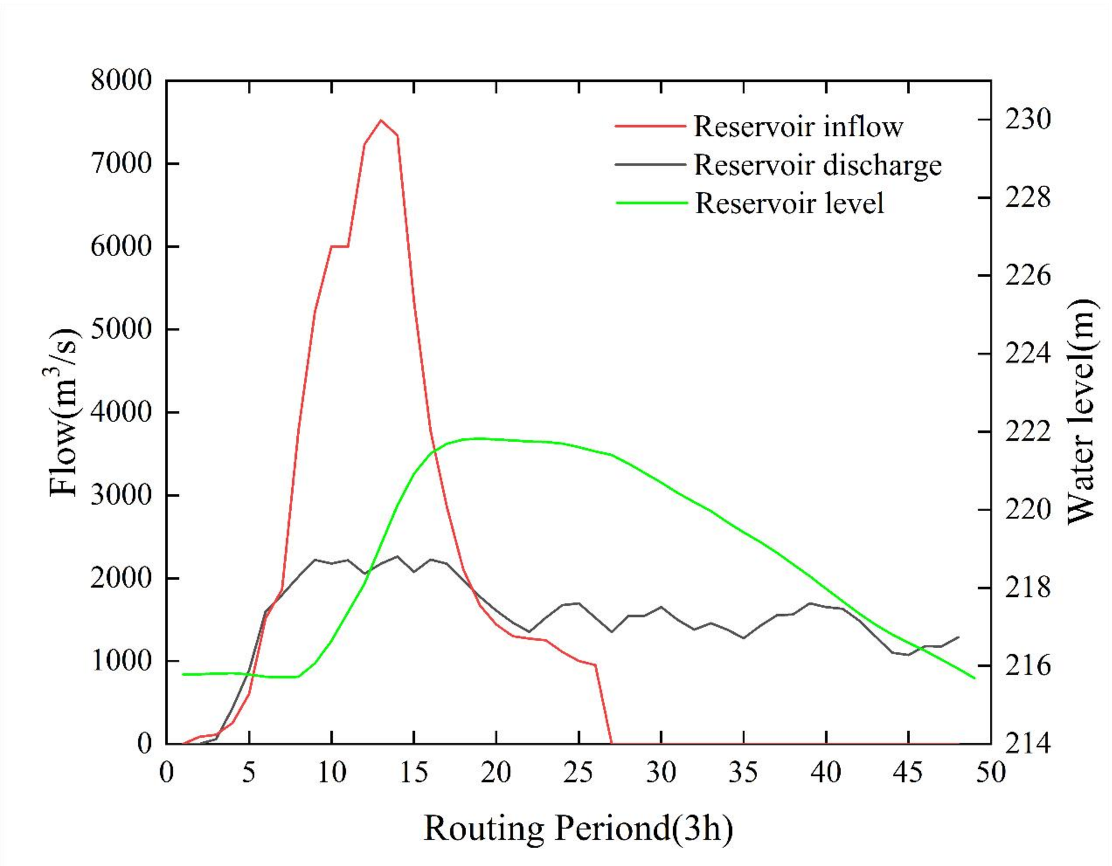


Fig 1. Flooding process in Panjia Kou

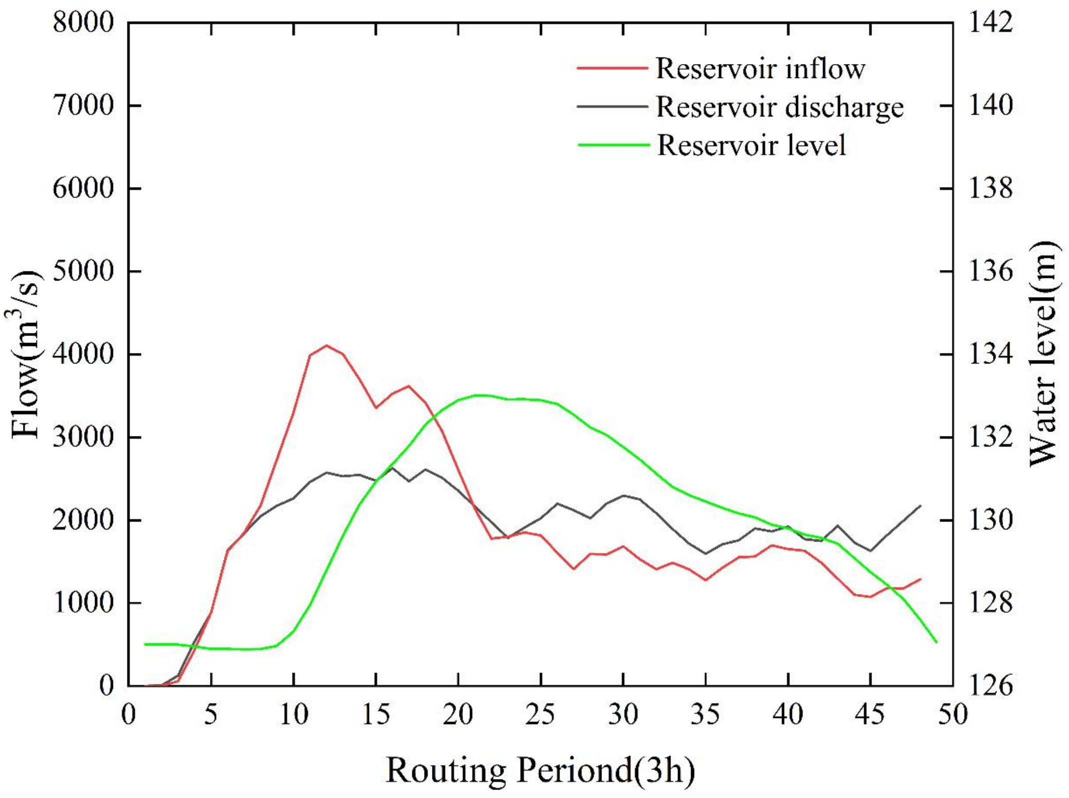


Fig 2. Process of flooding in Dahei Ting

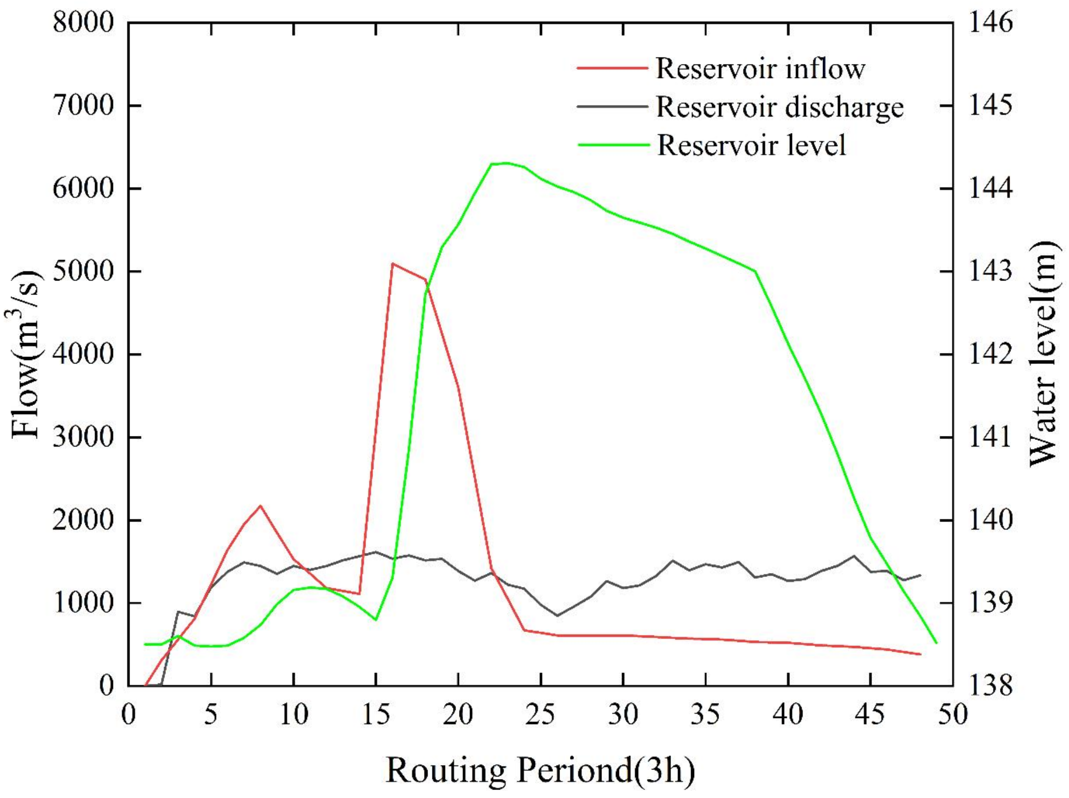


Fig 3. Process of flooding in Taolin Kou

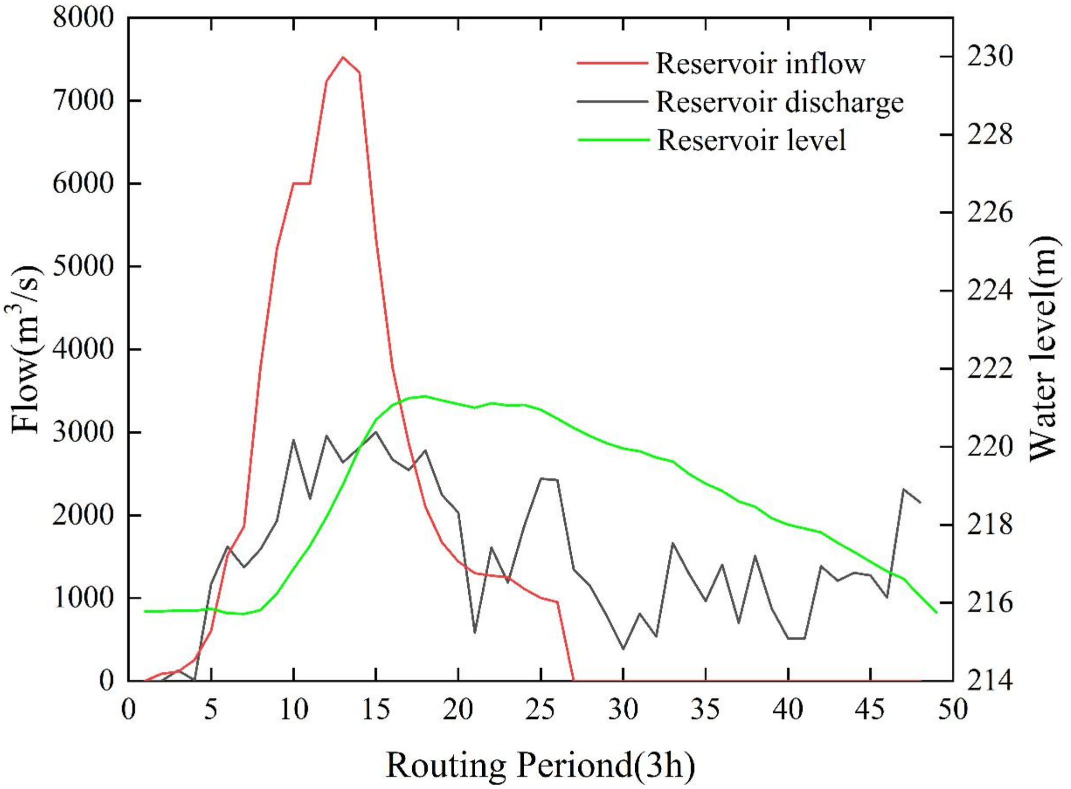


Fig 4. Flooding process in Panjia Kou(ε-DE)

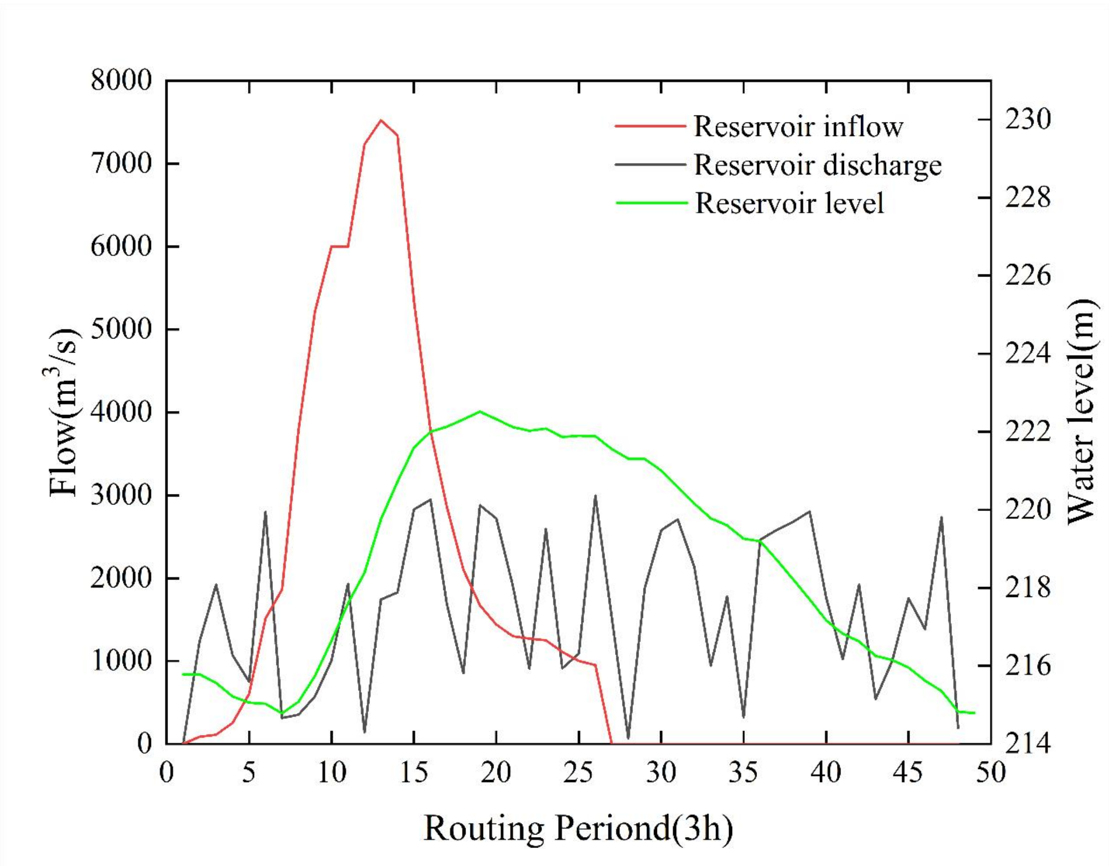


Fig 5. Flooding process in Panjia Kou(ε-WOA)

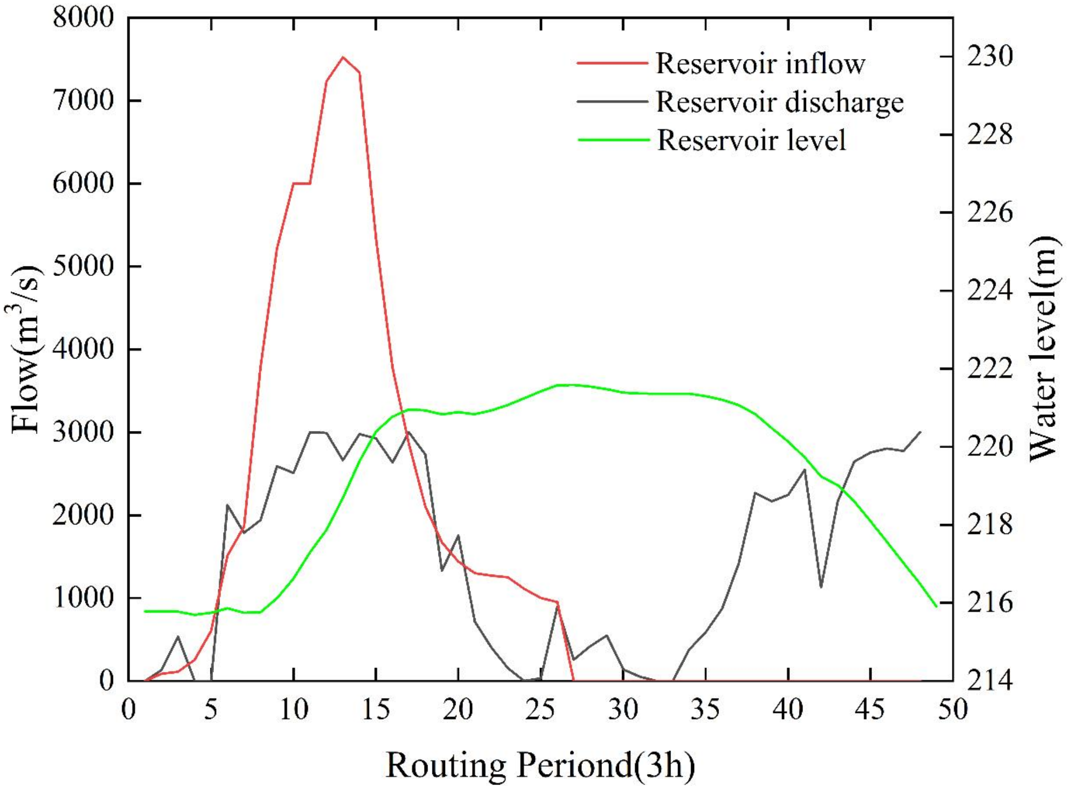


Fig 6. Flooding process in Panjia Kou(ε-PSO)
